# Comparison of spirometry, impulse oscillometry, and multiple breath washout in children with primary ciliary dyskinesia

**DOI:** 10.3389/fped.2026.1752410

**Published:** 2026-02-26

**Authors:** Utku Batu, Ela Erdem Eralp, Cansu Yılmaz Yeğit, Mine Kalyoncu, Mürüvvet Yanaz, Almala Pınar Ergenekon, Yasemin Gökdemir, Bülent Karadağ

**Affiliations:** 1 Department of Pediatrics, Van Regional Training and Research Hospital, Van, Türkiye; 2 Department of Pediatric Pulmonology, Marmara University, Istanbul, Türkiye; 3 Department of Pediatric Pulmonology, Kartal Lutfi Kirdar City Hospital, Istanbul, Turkiye; 4 Department of Pediatric Pulmonology, Diyarbakir Children’s Hospital, Diyarbakir, Türkiye

**Keywords:** impulse oscillometry, lung function, multiple breath washout, primary ciliary dyskinesia, spirometry

## Abstract

**Background:**

Primary ciliary dyskinesia (PCD) is associated with ventilation defects and heterogeneous impairment of pulmonary function. Spirometry alone may underestimate PCD severity and complexity. This study aimed to evaluate spirometry, multiple breath washout (MBW), and impulse oscillometry (IOS) in children with PCD and healthy controls.

**Methods:**

In this cross-sectional, prospective study, participants included children aged 6–18 years with PCD and healthy age-matched controls. Pulmonary function tests using MBW, IOS, and spirometry were conducted on the same day for all participants.

**Results:**

Thirty-two children with PCD (cwPCD) (median age 16.5 years) and 44 age-matched healthy controls (median age 15.7 years) were studied. PCD was associated with lower forced expiratory volume in 1 (FEV1) percent predicted (pp), forced vital capacity (FVC) pp, FEV1/FVC, reactance 5 (X5); as well as higher resistance 5 (R5), R10, R15, R20, resonance frequency (Fres) and lung clearance index (LCI) 2.5% mean values (*p* < 0.05 for all). Abnormal LCI 2.5% was found in 46.5% of patients with predicted FEV1 pp > 80%. Significant inverse correlations were observed between LCI 2.5% and FEV1 pp (*p* < 0.001, r: −0.62), FVC pp (*p* = 0.004, r: −0.49), FEV1/FVC (*p* = 0.002, r: −0.52) in PCD patients.

**Conclusion:**

This is one of the few studies comparing MBW, IOS, and spirometry in cwPCD. The study has shown that there are significant differences in spirometry and MBW between cwPCD and healthy controls. MBW can detect airway anomalies earlier than spirometry and may be used in follow-up as an alternative pulmonary function test in cwPCD.

## Introduction

Primary ciliary dyskinesia (PCD) is a rare disease caused by defects in the structure and/or function of motile cilia, resulting in impaired mucociliary clearance and recurrent airway infections (1). Early diagnosis, treatment, and close monitoring are crucial for preventing lung damage and preserving lung function (2).

There is a heterogeneity in lung function among patients with PCD. Spirometry is typically used to monitor patients with chronic lung diseases such as PCD and cystic fibrosis (CF). Although PCD was once thought to be a milder disease with slower progression than CF, studies have shown that lung function in people with PCD can range from normal to severely impaired. A recent meta-analysis revealed significant differences in forced expiratory volume in 1 (FEV1) percent predicted (pp) and forced vital capacity (FVC) pp measurements, with abnormal FEV1 pp values detected at the age of 6, demonstrating that the lung is affected early in PCD (3, 4). However, spirometry may not be highly sensitive in detecting mild-to-moderate lung damage in children with PCD (cwPCD), as it only assesses large airway flow and resistance and cannot evaluate the peripheral airways (5).

Impulse oscillometric study (IOS) is a non-invasive airway mechanics test that requires minimal patient cooperation. It measures resistance and reactance values during tidal breathing and is sensitive to peripheral airway diseases (6). Although some studies have assessed its usefulness in pediatric respiratory diseases such as asthma and CF, there are only a few studies including cwPCD (7–9).

Multiple breath inert gas washout (MBW) is a lung function technique to measure ventilation inhomogeneity. The lung clearance index (LCI) is a common measure derived from MBW tests, and offers information on lung pathology complementary to that from conventional lung function tests such as spirometry. MBW is a valuable tool for assessing small airway dysfunction and ventilation inhomogeneity in children. It is performed during tidal breathing without requiring forced expiratory maneuvers and is more sensitive than spirometry for detecting early peripheral airway disease in young children (10). However, conflicting data exist regarding the relationship between FEV1 pp and MBW in people with PCD, as several studies have demonstrated abnormal MBW results despite normal spirometric values (11, 12).

Our primary aim was to evaluate the relationship among spirometry, IOS, and MBW in cwPCD. The secondary objective was to compare the pulmonary function test results of cwPCD with those of healthy controls using these three methods. We hypothesized that IOS and MBW could identify lung function impairment earlier than spirometry in cwPCD.

## Materials and methods

A prospective, cross-sectional, single-center study was conducted between September 2019 and November 2020, which was approved by the Research Ethics Committee of Marmara University (approval number: 09.2019.802). Parental and participant informed consent was obtained, including for those over 12 years of age.

### Study design

This study enrolled cwPCD aged 6–18 years, along with age-matched healthy control children. The diagnosis of PCD was determined according to the algorithm of the European Respiratory Society (ERS). The diagnosis of PCD was based on non-certain biallelic mutations in known PCD-associated genes, electron microscopic evaluation of nasal cilia biopsy, positive immunofluorescent staining, and decreased nasal nitric oxide level according to ERS (13). All patients had clinical features consistent with PCD, and alternative diagnoses were excluded. The control group comprised healthy children with normal growth and development. Children with acute respiratory infections or evidence of pulmonary exacerbation were excluded from both groups. Demographic, clinical, laboratory, and radiological findings were obtained from medical records.

### Pulmonary function tests

Pulmonary function evaluation commenced with IOS, followed by MBW, and was concluded with spirometry.

#### Spirometry

Spirometry (MIR Winspro PRO 2.8, Rome, Italy) was conducted according to the international guidelines for interpretation while children were seated and awake (14). The measurements obtained included FVC pp, FEV1 pp, FEV1/FVC, forced expiratory flow (FEF) 75%, and FEF 25%–75%. The best of the three acceptable measurements was selected. A minimum of 80% of the predicted FEV1 pp and FVC pp values were considered normal according to preserved ratio impaired spirometry.

#### Impulse oscillometry

The children underwent IOS (Vyntus® IOS) to evaluate the input impedance of the respiratory system. This was achieved by generating small pressure oscillations transmitted to the lungs. Measurements were obtained every 30 s while the children were seated upright, breathing quietly with their heads in a neutral position through a mouthpiece. The IOS parameters, including the resistance (R), reactance (X), impedance (Z), resonance frequency (Fres), and reactance area (AX), were recorded. Xand R parameters were measured at 5, 10, 15, and 20 Hz. A large airway resistance is reflected by the resistance at high frequencies, while the overall airway resistance is reflected by the resistance at low frequencies. The small airway resistance is reflected in the difference in the resistance between 5 and 20 Hz. The coherence values, which reflect the reliability of the IOS measurements, were evaluated for 30 s of testing.

Acceptable coherence values were ≥0.6 at 5 Hz and ≥0.8 at 10 Hz. At least three tests were performed for each child and the best value was used for analysis (15).

#### Multiple breath washout

MBW test was performed by using Exhalyzer D, EcoMedics AG, Switzerland (version 3.2.1) (Nitrogen-based) according to current guidelines. The MBW test represents the number of functional residual capacity (FRC) turnovers needed to reduce the inert tracer gas concentration in the lungs to 1/40th (2.5%) and 1/20th (5%) of the initial level. Only trials that passed quality control according to the ERS Consensus Criteria were considered as successful (16). The final MBW test value was the average ± standard deviation (SD) of all the acceptable measurements for LCI 2.5% and LCI 5%. The normal values for LCI 2.5% and IOS parameters were determined according to the mean ± SD value of the results of the healthy control group.

## Statistical analysis

The study utilized SPSS 21.0 (IBM) programs to analyze the results. Descriptive statistics were used to summarize the data. The normality of the numerical variables was visually inspected using the Kolmogorov–Smirnov/Shapiro–Wilk test. Chi-square test was used to determine the relationships between categorical variables. Independent sample *t*-test was used to compare the means of the groups if the data were normally distributed. If normality was not provided, the Mann–Whitney *U*-test was used. Pearson's correlation test was used to assess whether the groups were normally distributed. If normality was not provided, the Spearman correlation test was used. A *p*-value of less than 0.05 was considered statistically significant.

## Results

### Demographic and clinical characteristics of the group

The study included 32 cwPCD (59.4%, *n* = 19 girls) who were followed up at the Marmara University Division of Pediatric Pulmonology and 44 healthy children (52.2%, *n* = 23 girls). There were no significant differences between the groups in terms of age, sex, BMI, height, or weight percentiles (*p* > 0.05 for all). Table 1 summarizes the demographic and diagnostic findings of cwPCD.

**Table 1:** Demographic and diagnostic data of children with PCD.

Clinical records	PCD group (*n*:32)
Age (year), median (25–75p)	16.5 (14–20)
Male, *n* (%)	13 (40.6)
BMI, median (25–75p)	22.2 (19.4–25.7)
PICADAR score (mean ± SD)	7.8 (3.1)
Nasal nitric oxide (nl/min) median (25–75p)	15.2 (9.3–36.4)
Immunofluorescence analysis (*n*: 23)	
Normal, *n* (%)	3 (9.3)
DNAH5 mislocation, *n* (%)	5 (15.6)
DNAH5 absent, *n* (%)	4 (12.5)
RSPH9 mislocation, *n* (%)	3 (9.4)
Others, *n* (%)	8 (25)
High-speed video microscopy motion pattern (*n*: 25)	
Immotile, *n* (%)	14 (43.7)
Abnormal ciliary pattern, *n* (%)	11 (34.3)
Genetic results	
CCDC40 homozygous, *n* (%)	6 (18.7)
DNAH5 homozygous, *n* (%)	6 (18.7)
CCNO homozygous, *n* (%)	2 (6.2)
RSPH4A homozygous, *n* (%)	2 (6.2)
DNAH11 homozygous, *n* (%)	1 (3.1)
Others, *n* (%)	16 (50)

BMI, body mass index.

### Pulmonary function tests

According to spirometry, control group had significantly higher mean values for FEV1 pp, FVC pp, FEV1/FVC, FEF50 pp, FEF25 pp, and FEF25-75 pp (*p* < 0.05 for all) than cwPCD. Mean values of R5, R10, R15, R20, and Fres values of the controls were significantly lower than those of the PCD group (*p* < 0.05 for all), whereas the LCI 2.5% and LCI 5% values were significantly higher in cwPCD (*p* < 0.05 for both) (Table 2).

**Table 2:** Comparison of lung function tests: spirometry, MBW and IOS results of children with PCD and control group.

Parameters	PCD patients (*n* = 32) (mean ± SD)	Control Group (*n* = 44) (mean ± SD)	*p*-value
Spirometry			
FEV_1_ pp (% predicted)	85.7 ± 21.5	104.3 ± 10	**<0** **.** **001**
FVC pp (% predicted)	87.7 ± 17	99.5 ± 11.4	**<0** **.** **001**
FEV_1_/FVC	97.7 ± 10.3	105.2 ± 8	**0** **.** **001**
FEF_75_ pp (% predicted)	81.7 ± 31.9	92.1 ± 20.4	0.08
FEF_50_ pp (% predicted)	75.2 ± 35.5	97.6 ± 21.8	**0** **.** **001**
FEF_25_ pp (% predicted)	57.9 ± 35	94 ± 32.3	**<0** **.** **001**
FEF_25-75_ pp (% predicted)	71.4 ± 34.8	97.7 ± 21.5	**<0** **.** **001**
IOS			
R5 Hz kPa(L/s)	0.6 ± 0.2	0.5 ± 0.2	**0** **.** **04**
R10 Hz kPa(L/s)	0.5 ± 0.1	0.4 ± 0.1	**0** **.** **01**
R15 Hz kPa(L/s)	0.4 ± 0.1	0.3 ± 0.1	**0** **.** **007**
R20 Hz kPa(L/s)	0.4 ± 0.1	0.3 ± 0.1	**0** **.** **006**
X5 Hz kPa(L/s)	−0.16 ± 0.06	−0.12 ± 0.05	**0** **.** **001**
X10 Hz kPa(L/s)	−0.1 ± 0.06	−0.08 ± 0.07	0.41
X15 Hz kPa(L/s)	−0.51 ± 0.05	−0.03 ± 0.06	0.27
X20 Hz kPa(L/s)	0.02 ± 0.04	0.04 ± 0.04	0.08
Fres (1/s)	18 ± 3.3	16.2 ± 3.7	**0** **.** **04**
MBW			
LCI 2.5%	10.9 ± 3.3	7 ± 0.7	**<0** **.** **001**
LCI 5%	6.4 ± 1.4	5 ± 0.3	**<0** **.** **001**

FEV1, forced expiratory volume in 1 s; FVC, forced vital capacity; FEF, forced expiratory flow; R, resistance; X, reactance; Fres, resonance frequency; IOS, impulse oscillometry; MBW, multiple breath washout; LCI, lung clearance index. The bold values indicated that were statistically significant *p* < 0.05.

When the FEV1pp and LCI 2.5% results were compared, 46.5% of cwPCD had high LCI 2.5% values even when FEV1 pp was normal, and this difference was statistically significant (*p* = 0.001). However, no statistically significant correlation was observed between FEV1 pp and R5-X5 values in cwPCD (Table 3).

**Table 3:** Distribution of LCI, R5, X5 and FEV1 values in children with PCD.

Parameters	LCI 2.5% > 7		LCI 2.5% < 7		*p*
	*n*	%	*n*	%	
FEV1 pp ≥ %80	15	%46.5	5	%15.6	**0** **.** **001**
FEV1 pp < %80	11	%34.3	1	%0.3	
	R5 > 0.5		R5 < 0.5		*p*
	*N*	%	*N*	%	
FEV1 pp ≥ %80	9	%28.1	9	%28.1	0.234
FEV1 pp < %80	4	%12.5	10	%31.2	
	X5 > −0.12		X5 < −0.12		*p*
	*N*	%	*N*	%	
FEV1 pp ≥ %80	8	%25	7	%21.8	0.828
FEV1 pp < %80	8	%25	9	%28.1	

FEV1, forced expiratory volume in 1 s; FVC, forced vital capacity; X, reactance; R, resistance; LCI, lung clearance index. The bold values indicated that were statistically significant *p* < 0.05.

In cwPCD; FEV1 pp and FVC pp were found to be positively correlated with X15, X20 (*p*: 0.04, r: 0.35; *p*: 0.01, r: 0.43; *p*: 0.04, r: 0.36; *p*: 0.02, r: 0.40; respectively), whereas FEV1 pp and FVC pp were negatively correlated with Fres, LCI 2.5% and LCI 5% (*p*: 0.006, r: −0.47; *p*: <0.001, r: 0,62; *p*: 0.001, r: −0.54; *p*: 0.01, r: −0.43; *p*: 0.004, r: −0.49; *p*: 0.02, r: −0.4; respectively) (Table 4).

**Table 4:** Correlation between spirometry, LCI and IOS results in children with PCD.

Variables (p/r)	FEV1 pp	FVC pp	FEV1/FVC pp	FEF75 pp	FEF25-75 pp
R5	0.656	0.403	0.414	0.347	0.463
	−0.082	−0.153	0.15	−0.172	−0.135
R10	0.974	0.844	0.449	0.509	0.614
	0.006	−0.036	0.139	−0.121	−0.093
R15	0.774	0.875	0.5	0.628	0.76
	0.053	0.029	0.124	−0.089	−0.056
R20	0.582	0.722	0.383	0.715	0.996
	0.101	0.065	0.16	−0.067	0.001
X5	0.31	0.55	0.29	0.29	0.07
	0.18	0.1	0.19	0.19	0.31
X10	0.11	0.07	0.9	0.14	0.09
	0.28	0.32	0.02	0.26	0.3
X15	**0** **.** **04**	**0** **.** **04**	0.57	0.05	**0** **.** **02**
	**0** **.** **35**	**0** **.** **36**	0.1	0.33	**0** **.** **38**
X20	**0** **.** **01**	**0** **.** **02**	0.24	**0** **.** **02**	**0** **.** **009**
	**0** **.** **43**	**0** **.** **4**	0.21	**0** **.** **39**	**0** **.** **45**
Fres	**0** **.** **006**	**0** **.** **01**	**0** **.** **01**	**0** **.** **009**	**0** **.** **002**
	**−0** **.** **47**	**−0** **.** **43**	**−0** **.** **22**	**−0** **.** **45**	**−0** **.** **52**
LCI 2.5%	**<0** **.** **001**	**0** **.** **004**	**0** **.** **002**	0.13	**0** **.** **002**
	**−0** **.** **62**	**−0** **.** **49**	**−0** **.** **52**	−0.27	**−0** **.** **52**
LCI 5%	**0** **.** **001**	**0** **.** **02**	**0** **.** **002**	0.13	**0** **.** **004**
	**−0** **.** **54**	**−0** **.** **4**	**−0** **.** **52**	−0.26	**−0** **.** **49**

FEV1, forced expiratory volume in 1 s; FVC, forced vital capacity; FEF, forced expiratory flow; R, resistance; X, reactance; Fres, resonance frequency; LCI, lung clearance index. The bold values indicated that were statistically significant *p* < 0.05.

## Discussion

Our study demonstrates that IOS and MBW tests are valuable pulmonary function tests that can complement or even serve as alternatives to spirometry in cwPCD. Consistent with previous studies, our findings support that MBW can detect lung damage earlier than spirometry in PCD (17, 18). To our knowledge, there is only one study that utilized a combination of IOS, MBW, and spirometry in cwPCD, highlighting the unique contribution of our study (19). But there was no study comparing IOS, MBW, and spirometry in both cwPCD and healthy control groups.

Spirometry is a test that measures lung function by testing airway resistance with forced inspiration and expiration. We found that FEV1 pp, FVC pp, FEV1/FVC, FEF25 pp, FEF50 pp, FEF25-75 pp and FEF75 pp values of the cwPCD were significantly lower than healthy controls. Similar findings were also reported in a large multicenter cohort study of 991 pediatric and adult PCD patients confirmed that mean FEV1 pp and FVC pp values were below reference ranges across age groups (20). Even though the mean spirometric values of the cwPCD are lower than healthy control group, they were within normal limits, with a mean FEV1 pp of 85.65 in our study. Similarly, multiple studies have reported normal spirometric values in cwPCD. Davis et al. reported median FEV1 pp as 89% in their study, including 118 cwPCD, while Moglione et al. reported mean FEV1 pp as 82.48%, noting a negative correlation between age at diagnosis and FEF25-75 pp values (21, 22). Another study compared people with CF and PCD, found lower FEF25-75 pp values in cwPCD, while there were no differences in terms of FEV1, FVC, and FEV1/FVC values, indicating early distal airway impairment in PCD, which spirometry alone may not detect (23). In addition, a high intrasubject variability in FEV1 pp was described in cwPCD patients due to the variable obstruction caused by secretions (17). These findings support the need for alternative diagnostic tests to improve the awareness of early lung function deterioration.

We found that R5, R10, R15, R20, and Fres values were significantly lower in healthy children, while X5 was significantly higher. There was no significant difference in terms of X10, X15 and X20 values between cwPCD and healthy controls. The absence of significant differences in higher-frequency reactance parameters (X10, X15, X20) may be explained by their predominant reflection of proximal airway mechanics. Given the relatively preserved lung function in our cohort, central airway involvement may have been limited at this stage of disease, resulting in unchanged IOS parameters reflecting proximal airways. When we compared spirometry and IOS results in cwPCD, we found that FEV1 pp with X15, X20, Fres; FVC pp with X15, X20, Fres, and FEV1/FVC with Fres were significantly correlated. Guan et al. reported that IOS, particularly Fres, effectively distinguishes bronchiectasis patients from healthy controls, as it reflects peripheral airway damage (24). Our study also found Fres to be positively correlated with all spirometric values, with cwPCD having higher Fres than controls. Studies on CF have shown comparable results with lower R5 and Fres values and higher X5 values in healthy controls (25). To our knowledge, there is only one study evaluating IOS in cwPCD. Gut et al. identified small airway dysfunction in 27 cwPCD using IOS, which showed a negative correlation with quality-of-life domains. This association was not observed with MBW or spirometry, suggesting that IOS may be more sensitive in detecting small airway involvement in cwPCD (19). In our study, 28.1% of patients had elevated R5 and 21.8% had decreased X5 despite normal FEV1pp values; however, these differences were not statistically significant. Postek et al. suggested that MBW may be more sensitive in detecting early lung abnormalities in CF; however, MBW and IOS assess different physiological aspects of lung function, with MBW reflecting ventilation inhomogeneity and IOS airway mechanics (26). IOS requires only tidal breathing without forced maneuvers, which makes it an easy, alternative test for small children or those who are unable to follow commands. However, given the uncertainties in IOS use and interpretation in PCD, larger, long-term studies are needed.

We evaluated the MBW test as a measure of global ventilation and small airway function in patients with PCD. Koucky et al. showed that ventilation inhomogeneity is significantly higher in cwPCD and CF compared to asthma (27). In our study, MBW test values at the 2.5% and 5% levels were significantly higher in cwPCD group compared to healthy controls, consistent with prior studies (11, 12). A significant negative correlation was observed between MBW values and FEV1 pp among cwPCD, which is also consistent with previous studies. Studies have shown that MBW test detects changes in lung function earlier than spirometry. Roehmel et al. reported that LCI was increased in preschool cwPCD and CF similarly, compared to healthy controls, and reported that lung disease severity may be similar to CF during preschool years in cwPCD (28). Zafar et al. found that MBW and CT findings became abnormal before FEV1 pp or FEF25-75 pp values in cwPCD (11). Nyilas et al. found that 52% of people with PCD had abnormal MRI and MBW test values despite normal FEV1 pp values, emphasizing the need for additional lung function tests to assess early lung injury (12). From a clinical perspective, MBW may be considered during routine follow-up of patients with PCD, particularly in those with normal spirometric indices, as LCI can detect early ventilation inhomogeneity not captured by FEV1 pp (29). Irving et al. reported that, while HRCT and MBW results were consistent, this condition was not observed with FEV1 pp in children with non-CF bronchiectasis (18). Our study showed that 46.5% of cwPCD had an increased LCI of 2.5% with normal FEV1 pp values, consistent with previous studies. However, a systematic review by Hine et al. highligted the need for high-quality studies to assess the role of MBW in children with bronchiectasis (30). To determine MBW's precise clinical role in PCD, further research with a larger sample size is necessary.

The current study had some limitations. First, it was a single-center study with a relatively small sample size. Long-term follow-up and repeated pulmonary function tests are necessary to determine the importance of these tests in disease follow-up. Additionally, there are no standard reference values for MBW and IOS for different populations, and more research with a larger sample size is needed. Moreover, we did not compare pulmonary function test results with radiological findings, including bronchiectasis severity. Lastly, the study did not include patients younger than 6 years of age, as MBW and IOS require tidal breathing, which may be more helpful than spirometry in younger children.

## Conclusion

This study compared children with PCD to healthy controls using spirometry, IOS, and MBW. MBW was particularly effective in identifying ventilation inhomogeneity, including in cases where spirometric values remained within the normal range. IOS provided complementary information on airway mechanics and small airway involvement, supporting its role as a valuable non-invasive assessment tool. Together, these findings highlight the benefit of incorporating MBW and IOS alongside conventional spirometry in the evaluation and follow-up of children with PCD. Further longitudinal, multicenter and large-scale studies with international collaborations are warranted to confirm their clinical utility and establish standardized reference values for these tests in cwPCD.

## Data Availability

The raw data supporting the conclusions of this article will be made available by the authors, without undue reservation.
